# Supplementary material for: Antibiotic Susceptibility and Resistance Genes in Oral Clinical Isolates of Prevotella intermedia, Prevotella nigrescens, and Prevotella melaninogenica
Source: Antibiotics (Basel). 2022 Jul 4;11(7):888. doi: 10.3390/antibiotics11070888 (PMC9312306; doi:10.3390/antibiotics11070888)
Supplement: Supplementary file 1 [file antibiotics-11-00888-s001.zip › antibiotics-1774972-supplementary.pdf]

**S1**. Prevalence of resistance genes in *P. intermedia, P . nigrescens* and *P. melaninogenica*

The prevalence of genes for resistance on the 162 isolates to β- lactam antibiotics such as *cfxA* , *cfxA2*, *bla$_{TEM}$*, tetracyclines such as *tetM* , *tetQ* , macrolides such as *ermF* , and nitroimidazoles such as *nimAB* and *nimAEFI* was determined for isolates of *P. intermedia, P. nigrescens* and *P. melaninogenica.* The gene most frequently detected was *tetQ* in 40.7%, followed by *cfxA* in 26.5%, *tetM in* 25%, *ermF in* 20.6%, *bla $_{TEM}$* in 17.9%, and *nimAB in a lower percentage.* with 9.4%, *cfxA2* , with 5%, and *nimAEFI* with 1.2%.

In *P. intermedia* , the tetracycline resistance gene *tetQ* was detected more frequently in 44.9%, followed by *tetM* in 34.7%, and less frequently *cfxA* in 16.3%, *ermF* in 6.1%, *bla$_{TEM}$* in 4.1%, and *nimAB* in 2%. *nimAEFI* was not detected. For *P. nigrescens tetQ gene was detected in* 46%, *cfxA* 40.5%, *cfxA2* 1.3%, *ermF* 33.8%, *bla $_{TEM}$* 25.6%, *tetM* 20.3%, and less frequently *nimAB* 8.1%, and *nimAEFI* 1.3%. In *P. melaninogenica cfxA2* , *bla$_{TEM}$* , *tetM,* and *nimAB* were detected more frequently with 20.5%, and less frequently *cfxA* and e*rmF* with 12.8%, and *nimAEFI* with 2.6%.

**Table S1**. Frequency of genes c*fxA,* c*fxA2 , bla$_{TEM}$, tetM, tetQ, ermF, nimAB* and *nimAEFI* in 162 oral isolates of *P. intermedia, P. nigrescens,* y *P.melaninogenica*

| ***Bacteria*** | **n** | ***cfxA*** **F(%)** | ***cfxA2*** **F(%)** | ***bla$_{TEM}$*** **F(%)** | ***tetM*** **F(%)** | ***tetQ*** **F(%)** | ***nimAB*** **F(%)** | ***nimAEFI*** **F(%)** | ***ermF*** **F(%)** |
|---|---|---|---|---|---|---|---|---|---|
| *P. intermedia* | 49 | 8(16,3) | 0 | 2(4,1) | 17(34,7) | 22(44,9) | 1(2) | 0 | 3(6,1) |
| *P. nigrescens* | 74 | 30(40,5) | 1(1.3) | 19(25,6) | 15(20,3) | 34(46) | 6(8,1) | 1 (1,3) | 25(33,8) |
| *P. melaninogenica* | 39 | 5(12,8) | 8(20,5) | 8(20,5) | 8(20,5) | 11(28,2) | 8(20,5) | 1(2,6) | 5(12,8) |
| *Total* | 162 | 43(26,5) | 8 (5) | 29(17,9) | 40 (25) | 66(40,7) | 15 (9,4) | 2 (1,2) | 33(20,6) |
